# Supplementary material for: Divergence and Remarkable Diversity of the Y Chromosome in Guppies
Source: Mol Biol Evol. 2020 Oct 6;38(2):619–33. doi: 10.1093/molbev/msaa257 (PMC7826173; doi:10.1093/molbev/msaa257)

Supplementary Information

Supplementary Table S1. Sequencing information for each sample.

					Raw data	Filtered data		
**Sample**	**Sex**	**River**	**Population**	**Method**	**# reads**	**# reads**	**% reads**	**Coverage***
ArH-F1	Female	Aripo	Downstream	Illumina	177,001,862	176,505,596	99.72	28.97
ArH-F9	Female	Aripo	Downstream	Illumina	123,686,724	123,461,396	99.82	16.83
ArH-F10	Female	Aripo	Downstream	Illumina	139,802,356	139,241,328	99.60	22.89
ArH-F13	Female	Aripo	Downstream	Illumina	105,446,352	105,237,508	99.80	17.27
ArH-F14	Female	Aripo	Downstream	Illumina	163,351,722	162,980,354	99.77	22.89
ArH-F23	Female	Aripo	Downstream	Illumina	191,928,134	191,596,890	99.83	31.57
ArH-F25	Female	Aripo	Downstream	Illumina	109,682,202	109,408,568	99.75	17.94
ARH-F2	Female	Aripo	Downstream	10x	408,970,038	402,683,964	98.46	37.71
ArH-F11	Female	Aripo	Downstream	10x	381,405,262	368,721,272	96.67	32.21
ARH-F24	Female	Aripo	Downstream	10x	426,713,902	420,185,200	98.47	38.33
ARH-M1	Male	Aripo	Downstream	10x	439,226,730	432,609,612	98.49	39.32
ArH-M2	Male	Aripo	Downstream	10x	252,352,686	246,637,432	97.74	26.50
ArH-M5	Male	Aripo	Downstream	10x	260,888,232	256,042,222	98.14	23.71
ArH-M7	Male	Aripo	Downstream	10x	355,642,886	348,246,874	97.92	35.55
ArH-M9	Male	Aripo	Downstream	10x	387,869,768	380,303,506	98.05	40.35
ArH-M12	Male	Aripo	Downstream	10x	288,489,532	283,211,948	98.17	26.85
ARH-M16	Male	Aripo	Downstream	10x	466,170,214	459,951,168	98.67	45.05
ArH-M19	Male	Aripo	Downstream	10x	485,992,400	471,350,870	96.99	35.21
ArH-M21	Male	Aripo	Downstream	10x	241,425,146	235,678,560	97.62	23.68
ArH-M24	Male	Aripo	Downstream	10x	302,563,204	295,221,194	97.57	26.84
ArL-F5	Female	Aripo	Upstream	Illumina	125,681,718	125,115,518	99.55	21.00
ArL-F8	Female	Aripo	Upstream	Illumina	145,627,362	145,164,072	99.68	24.14
ArL-F9	Female	Aripo	Upstream	Illumina	146,020,928	145,499,770	99.64	24.39
ArL-F15	Female	Aripo	Upstream	Illumina	139,996,196	139,510,334	99.65	23.47

ArL-F18	Female	Aripo	Upstream	Illumina	149,839,898	149,483,814	99.76	24.85
ArL-F19	Female	Aripo	Upstream	Illumina	129,400,664	128,984,726	99.68	21.64
ArL-F20	Female	Aripo	Upstream	Illumina	121,795,694	121,199,836	99.51	20.19
ArL-F10	Female	Aripo	Upstream	10x	331,020,806	326,156,754	98.53	30.38
ArL-F16	Female	Aripo	Upstream	10x	337,717,026	332,234,258	98.38	29.81
ArL-F22	Female	Aripo	Upstream	10x	281,639,728	275,613,302	97.86	26.56
ArL-M1	Male	Aripo	Upstream	10x	395,101,320	385,055,732	97.46	34.29
ArL-M6	Male	Aripo	Upstream	10x	344,463,254	336,457,206	97.68	29.02
ArL-M11	Male	Aripo	Upstream	10x	374,115,442	364,106,466	97.32	31.81
ArL-M12	Male	Aripo	Upstream	10x	307,371,902	296,634,782	96.51	27.48
ARL-M15	Male	Aripo	Upstream	10x	385,877,220	380,935,470	98.72	37.81
ArL-M16	Male	Aripo	Upstream	10x	369,068,090	360,681,320	97.73	35.27
ARL-M17	Male	Aripo	Upstream	10x	395,209,344	389,830,668	98.64	37.37
ArL-M19	Male	Aripo	Upstream	10x	354,769,736	346,126,668	97.56	28.64
ArL-M24	Male	Aripo	Upstream	10x	316,277,460	309,367,708	97.82	32.45
ARL-M25	Male	Aripo	Upstream	10x	348,601,062	343,193,820	98.45	32.86
QuH-F4	Female	Quare	Downstream	Illumina	179,052,012	178,453,836	99.67	28.62
QuH-F6	Female	Quare	Downstream	Illumina	106,006,310	105,684,712	99.70	17.02
QuH-F7	Female	Quare	Downstream	Illumina	146,614,088	146,074,580	99.63	25.19
QuH-F9	Female	Quare	Downstream	Illumina	125,867,070	125,295,106	99.55	21.45
QuH-F12	Female	Quare	Downstream	Illumina	147,284,914	146,610,000	99.54	24.65
QuH-F13	Female	Quare	Downstream	Illumina	147,789,606	147,211,156	99.61	25.05
QuH-F22	Female	Quare	Downstream	Illumina	122,308,096	122,024,688	99.77	17.76
QuH-F3	Female	Quare	Downstream	10x	383,364,788	376,444,214	98.19	34.92
QuH-F21	Female	Quare	Downstream	10x	372,586,380	364,173,432	97.74	34.35
QuH-F25	Female	Quare	Downstream	10x	262,839,572	258,146,910	98.21	24.71
QuH-M2	Male	Quare	Downstream	10x	332,995,698	325,603,698	97.78	28.54
QuH-M3	Male	Quare	Downstream	10x	304,584,270	299,436,134	98.31	29.42

QUH-M4	Male	Quare	Downstream	10x	397,440,770	391,344,378	98.47	34.47
QUH-M9	Male	Quare	Downstream	10x	397,506,462	391,924,012	98.60	40.17
QuH-M10	Male	Quare	Downstream	10x	306,774,330	300,809,780	98.06	29.66
QuH-M13	Male	Quare	Downstream	10x	159,301,776	156,661,362	98.34	17.66
QuH-M17	Male	Quare	Downstream	10x	334,204,944	328,118,800	98.18	32.04
QuH-M19	Male	Quare	Downstream	10x	329,744,366	324,159,304	98.31	31.40
QuH-M22	Male	Quare	Downstream	10x	339,581,552	332,344,120	97.87	28.45
QuL-F4	Female	Quare	Upstream	Illumina	113,172,552	112,900,498	99.76	19.19
QuL-F6	Female	Quare	Upstream	Illumina	123,311,704	122,950,174	99.71	20.49
QuL-F7	Female	Quare	Upstream	Illumina	116,293,246	116,001,858	99.75	18.74
QuL-F14	Female	Quare	Upstream	Illumina	218,161,778	217,313,576	99.61	35.62
QuL-F15	Female	Quare	Upstream	Illumina	130,978,920	130,701,716	99.79	21.02
QuL-F19	Female	Quare	Upstream	Illumina	92,666,384	92,425,842	99.74	15.16
QuL-F23	Female	Quare	Upstream	Illumina	118,582,976	118,221,148	99.69	18.98
QUL-F5	Female	Quare	Upstream	10x	359,994,034	355,396,326	98.72	34.67
QuL-F11	Female	Quare	Upstream	10x	357,689,770	349,210,524	97.63	29.03
QUL-F16	Female	Quare	Upstream	10x	478,732,114	471,211,580	98.43	44.48
QuL-M2	Male	Quare	Upstream	10x	308,996,474	303,509,088	98.22	36.16
QUL-M4	Male	Quare	Upstream	10x	378,405,724	371,842,898	98.27	35.87
QuL-M5	Male	Quare	Upstream	10x	376,139,404	365,675,072	97.22	28.20
QuL-M6	Male	Quare	Upstream	10x	339,915,846	333,810,516	98.20	28.95
QuL-M8	Male	Quare	Upstream	10x	370,727,306	364,327,302	98.27	33.78
QuL-M11	Male	Quare	Upstream	10x	323,424,146	316,698,712	97.92	26.86
QuL-M15	Male	Quare	Upstream	10x	317,275,484	312,358,620	98.45	28.26
QuL-M17	Male	Quare	Upstream	10x	346,548,086	341,135,520	98.44	30.95
QuL-M20	Male	Quare	Upstream	10x	324,939,344	318,958,212	98.16	38.31
QuL-M25	Male	Quare	Upstream	10x	338,420,592	333,069,042	98.42	34.08
YaH-F9	Female	Yarra	Downstream	Illumina	154,552,664	154,130,620	99.73	21.32

YaH-F13	Female	Yarra	Downstream	Illumina	137,843,852	137,496,702	99.75	20.34
YaH-F14	Female	Yarra	Downstream	Illumina	142,201,016	141,656,306	99.62	19.50
YaH-F15	Female	Yarra	Downstream	Illumina	152,591,576	152,311,356	99.82	22.21
YaH-F18	Female	Yarra	Downstream	Illumina	130,309,702	129,898,602	99.68	19.13
YaH-F21	Female	Yarra	Downstream	Illumina	133,816,960	133,394,266	99.68	18.42
YaH-F24	Female	Yarra	Downstream	Illumina	122,636,722	122,188,898	99.63	16.68
YaH-F1	Female	Yarra	Downstream	10x	222,780,570	219,055,424	98.33	26.18
YAH-F12	Female	Yarra	Downstream	10x	356,192,978	350,570,380	98.42	34.30
YaH-F19	Female	Yarra	Downstream	10x	348,295,768	340,287,472	97.70	30.88
YaH-M2	Male	Yarra	Downstream	10x	236,620,736	231,584,160	97.87	23.60
YaH-M5	Male	Yarra	Downstream	10x	405,433,092	392,515,154	96.81	27.71
YaH-M6	Male	Yarra	Downstream	10x	298,369,238	282,064,166	94.54	19.68
YaH-M10	Male	Yarra	Downstream	10x	323,420,634	316,618,056	97.90	33.81
YaH-M11	Male	Yarra	Downstream	10x	295,137,132	286,247,122	96.99	21.57
YaH-M16	Male	Yarra	Downstream	10x	358,627,758	345,447,188	96.32	22.49
YaH-M17	Male	Yarra	Downstream	10x	328,438,330	316,504,572	96.37	23.90
YaH-M18	Male	Yarra	Downstream	10x	220,144,952	216,739,042	98.45	26.14
YaH-M21	Male	Yarra	Downstream	10x	326,897,482	315,901,204	96.64	23.42
YaH-M23	Male	Yarra	Downstream	10x	331,356,786	317,506,648	95.82	26.82
YaL-F4	Female	Yarra	Upstream	Illumina	138,514,074	138,110,048	99.71	20.81
YaL-F6	Female	Yarra	Upstream	Illumina	134,171,018	133,760,786	99.69	20.05
YaL-F10	Female	Yarra	Upstream	Illumina	117,695,078	117,470,256	99.81	20.12
YaL-F12	Female	Yarra	Upstream	Illumina	140,113,924	139,528,912	99.58	23.87
YaL-F19	Female	Yarra	Upstream	Illumina	139,747,920	139,451,146	99.79	20.42
YaL-F22	Female	Yarra	Upstream	Illumina	125,894,888	125,487,600	99.68	21.17
YaL-F24	Female	Yarra	Upstream	Illumina	125,153,320	124,768,922	99.69	17.14
YaL-F11	Female	Yarra	Upstream	10x	391,722,230	382,605,508	97.67	35.15
YaL-F13	Female	Yarra	Upstream	10x	307,344,588	300,997,862	97.93	28.00

YAL-F20	Female	Yarra	Upstream	10x	391,780,204	384,760,566	98.21	32.96
YaL-M7	Male	Yarra	Upstream	10x	363,941,054	357,078,016	98.11	32.47
YAL-M8	Male	Yarra	Upstream	10x	451,668,232	445,186,766	98.56	39.19
YAL-M10	Male	Yarra	Upstream	10x	494,223,386	483,699,440	97.87	40.37
YaL-M11	Male	Yarra	Upstream	10x	259,374,704	255,007,362	98.32	25.58
YaL-M14	Male	Yarra	Upstream	10x	409,708,944	398,129,722	97.17	29.28
YaL-M16	Male	Yarra	Upstream	10x	292,372,948	285,855,314	97.77	26.45
YaL-M17	Male	Yarra	Upstream	10x	267,080,378	262,237,792	98.19	26.12
YaL-M18	Male	Yarra	Upstream	10x	399,152,812	388,825,948	97.41	34.47
YaL-M21	Male	Yarra	Upstream	10x	368,029,652	360,600,418	97.98	34.95
YaL-M23	Male	Yarra	Upstream	10x	420,027,608	409,077,362	97.39	29.61
YaL-M25	Male	Yarra	Upstream	10x	332,547,264	325,775,770	97.96	29.89

* effective coverage of reference-guided alignments to river-specific reference genomes after read trimming and duplicate removal.

Supplementary Table S2. Assembly statistics for all 10x Genomics samples using Supernova *de-novo* assembler.

Sample	Sex	Source	Valid barcode %	Effective coverage	Molecule length	Assembly size	Contig N50	Scaffold N50	Scaffolds 10kb+	Scaffolds 1kb+	Phase N50
ArH-F2	Female	Ar-D	92.89	34.34	12,479	603,896,307	30,435	61,210	13,707	51,535	109,106
ArH-F11	Female	Ar-D	94.61	29.87	11,873	570,483,834	25,621	49,550	16,200	56,809	67,120
ArH-F24	Female	Ar-D	94.18	35.15	29,363	729,242,799	50,348	1,584,760	3,703	22,339	593,022
ArH-M1	Male	Ar-D	93.99	35.56	22,003	672,922,408	48,757	479,207	4,774	22,456	369,523
ArH-M2	Male	Ar-D	93.89	24.74	23,357	646,434,489	36,408	116,760	10,373	35,215	173,504
ArH-M5	Male	Ar-D	94.15	21.48	20,319	648,310,764	39,976	295,457	6,592	29,422	266,082
ArH-M7	Male	Ar-D	93.07	32.92	23,826	666,756,002	43,787	243,248	7,181	24,657	266,503
ArH-M9	Male	Ar-D	93.59	37.82	24,794	677,369,114	51,770	638,371	4,382	24,866	500,136
ArH-M12	Male	Ar-D	94.13	24.41	21,144	671,597,633	47,579	639,034	4,288	19,471	326,840
ArH-M16	Male	Ar-D	93.63	41.17	22,003	677,689,639	51,850	1,280,894	3,288	20,004	802,655
ArH-M19	Male	Ar-D	93.07	31.66	nd	84,351,704	15,431	16,164	5,101	132,798	2,874
ArH-M21	Male	Ar-D	93.32	20.96	16,325	597,049,153	26,596	48,636	16,778	54,789	67,539
ArH-M24	Male	Ar-D	94.35	24.94	9,926	600,957,212	28,856	45,523	17,703	49,508	56,221
ArL-F10	Female	Ar-U	93.86	28.00	22,003	682,875,263	54,076	1,518,110	3,022	14,584	557,061
ArL-F16	Female	Ar-U	93.78	26.84	33,418	683,530,463	54,203	3,333,357	2,517	13,828	1,079,421
ArL-F22	Female	Ar-U	94.26	23.90	20,118	667,305,062	42,648	345,876	5,857	22,437	267,979
ArL-M1	Male	Ar-U	94.25	31.45	18,032	670,441,308	40,452	173,277	8,260	25,177	176,037
ArL-M6	Male	Ar-U	94.01	26.32	23,357	675,835,125	43,709	357,112	5,849	20,424	263,802
ArL-M11	Male	Ar-U	94.44	29.05	24,794	673,017,375	42,428	229,772	6,736	22,192	206,621
ArL-M12	Male	Ar-U	94.67	25.33	11,074	621,217,985	28,539	47,766	17,259	41,614	55,199
ArL-M15	Male	Ar-U	93.09	33.94	26,848	685,292,827	52,226	2,842,056	2,832	16,074	887,754
ArL-M16	Male	Ar-U	94.42	32.45	21,356	686,717,238	50,777	883,946	3,690	15,732	541,151
ArL-M17	Male	Ar-U	92.92	33.62	14,061	670,913,526	44,741	211,057	6,905	22,347	162,071
ArL-M19	Male	Ar-U	93.91	25.35	13,512	616,636,301	29,873	54,820	15,075	40,051	64,261

ArL-M24	Male	Ar-U	93.75	30.23	6,869	580,898,802	29,105	42,628	17,901	49,183	55,408
ArL-M25	Male	Ar-U	94.05	30.24	20,319	676,980,460	51,386	725,226	3,906	17,368	415,239
QuH-F3	Female	Qu-D	94.02	32.20	12,233	615,355,903	30,730	59,296	14,154	50,604	105,695
QuH-F21	Female	Qu-D	94.38	32.08	9,166	574,798,522	28,246	43,776	17,691	59,624	59,614
QuH-F25	Female	Qu-D	94.13	24.74	28,500	641,956,330	39,791	319,579	7,006	28,515	307,182
QuH-M2	Male	Qu-D	93.95	25.98	5,858	295,245,989	15,868	19,569	15,930	112,140	25,216
QuH-M3	Male	Qu-D	93.55	26.69	11,639	559,237,759	28,649	48,359	16,102	61,755	66,441
QuH-M4	Male	Qu-D	93.47	30.45	23,826	669,172,328	50,682	778,580	3,698	19,469	454,672
QuH-M9	Male	Qu-D	93.56	36.89	18,032	673,996,478	53,012	791,350	3,736	19,521	432,210
QuH-M10	Male	Qu-D	94.10	27.49	11,755	558,402,613	26,999	49,136	15,908	64,502	77,392
QuH-M13	Male	Qu-D	94.32	16.29	24,064	577,989,203	26,391	137,986	9,804	48,823	161,594
QuH-M17	Male	Qu-D	93.81	29.38	16,325	645,193,812	37,249	101,325	10,804	38,131	152,354
QuH-M19	Male	Qu-D	93.71	28.59	11,410	552,950,240	27,686	48,242	16,282	64,242	74,587
QuH-M22	Male	Qu-D	93.62	25.76	7,438	546,134,524	25,575	36,669	18,916	61,646	52,960
QuL-F5	Female	Qu-U	92.81	30.86	35,474	685,901,629	53,492	5,195,008	1,935	9,980	752,633
QuL-F11	Female	Qu-U	93.98	25.90	5,251	453,708,191	19,440	23,168	21,506	73,073	13,022
QuL-F16	Female	Qu-U	94.13	40.02	16,163	682,908,655	53,788	732,948	3,061	11,812	301,380
QuL-M2	Male	Qu-U	93.63	34.01	26,058	689,830,587	61,318	2,298,331	2,071	9,801	510,678
QuL-M4	Male	Qu-U	94.05	32.64	26,848	681,774,619	55,402	2,186,889	2,101	11,233	563,437
QuL-M5	Male	Qu-U	94.08	25.24	4,390	355,020,979	17,467	18,886	19,401	87,904	4,001
QuL-M6	Male	Qu-U	93.98	26.16	25,545	674,390,728	48,692	579,889	3,951	13,599	300,025
QuL-M8	Male	Qu-U	94.27	31.43	21,785	685,637,933	56,036	922,322	2,921	11,240	327,493
QuL-M11	Male	Qu-U	94.17	24.32	11,755	632,789,117	32,662	54,421	15,250	34,413	60,085
QuL-M15	Male	Qu-U	93.81	25.39	25,800	680,690,864	53,789	1,703,422	2,525	11,868	406,911
QuL-M17	Male	Qu-U	94.01	27.94	26,582	687,822,904	56,629	2,561,222	2,242	11,099	524,879
QuL-M20	Male	Qu-U	93.86	35.85	38,797	692,284,958	58,647	4,694,176	1,941	10,097	806,503
QuL-M25	Male	Qu-U	93.82	31.27	36,187	688,491,763	59,049	4,135,504	1,999	10,226	760,282
YaH-F1	Female	Ya-D	93.77	24.84	26,058	662,631,832	48,239	1,215,823	3,303	18,049	364,287

YaH-F12	Female	Ya-D	93.42	32.61	27,387	670,254,389	45,107	549,157	5,056	20,604	306,641
YaH-F19	Female	Ya-D	94.30	28.78	4,613	216,742,755	14,626	16,251	13,267	116,625	4,554
YaH-M2	Male	Ya-D	94.47	21.87	7,438	514,720,685	25,053	36,636	18,026	64,522	46,584
YaH-M5	Male	Ya-D	94.39	24.49	13,922	661,982,631	33,347	78,737	12,837	36,672	116,081
YaH-M6	Male	Ya-D	94.58	17.94	nd	106,720,199	14,669	14,920	6,988	144,481	8,184
YaH-M10	Male	Ya-D	93.44	31.53	14,778	638,343,761	34,690	84,743	11,527	38,998	118,361
YaH-M11	Male	Ya-D	94.39	18.32	9,730	635,051,176	30,379	63,598	13,542	35,381	72,759
YaH-M16	Male	Ya-D	94.62	19.53	6,343	335,196,646	17,259	22,059	16,553	95,458	32,997
YaH-M17	Male	Ya-D	94.49	21.71	7,438	594,225,669	27,052	44,255	17,478	49,231	54,745
YaH-M18	Male	Ya-D	93.47	24.54	33,087	655,984,201	48,911	3,381,888	2,522	17,986	652,943
YaH-M21	Male	Ya-D	94.52	20.70	9,351	605,902,349	27,637	48,967	16,418	48,044	58,163
YaH-M23	Male	Ya-D	93.57	24.71	nd	133,227,602	15,422	15,974	8,145	131,841	3,866
YaL-F11	Female	Ya-U	94.10	33.73	4,849	360,358,685	17,971	21,695	17,977	93,589	11,515
YaL-F13	Female	Ya-U	93.45	25.51	5,686	394,307,353	19,417	23,452	18,662	89,176	11,138
YaL-F20	Female	Ya-U	93.54	29.50	3,974	433,524,338	17,204	19,105	23,613	73,137	5,377
YaL-M7	Male	Ya-U	93.53	29.53	6,035	561,508,794	28,237	41,876	17,766	51,394	55,289
YaL-M8	Male	Ya-U	92.27	34.70	10,432	607,100,629	33,158	63,066	13,121	41,571	86,745
YaL-M10	Male	Ya-U	94.12	36.13	4,390	355,606,474	16,423	19,660	19,152	92,067	4,784
YaL-M11	Male	Ya-U	93.65	23.32	12,479	609,683,092	33,128	61,585	13,338	41,236	68,388
YaL-M14	Male	Ya-U	93.87	26.68	nd	264,551,372	18,128	18,667	14,369	104,384	3,551
YaL-M16	Male	Ya-U	93.98	24.02	3,289	303,933,539	19,032	20,327	15,565	97,427	4,448
YaL-M17	Male	Ya-U	93.68	23.79	5,858	423,920,287	20,835	25,940	18,664	82,598	23,729
YaL-M18	Male	Ya-U	93.78	31.66	3,193	243,577,016	17,728	19,919	12,673	105,508	5,481
YaL-M21	Male	Ya-U	93.34	32.54	4,996	415,438,638	18,870	23,460	19,622	81,499	6,258
YaL-M23	Male	Ya-U	92.95	25.93	2,040	216,002,997	18,455	19,183	11,484	111,466	3,611
YaL-M25	Male	Ya-U	93.89	27.16	7,512	631,952,124	33,607	48,596	16,255	36,313	57,931

Ar = Aripo River, Ya = Yarra River, Qu = Quare River; D = Downstream/High-predation population, U = Upstream/Low-predation population
nd = Supernova could not determine molecule length, likely due to sub-optimal input DNA quality, but the assembly process was successful.

Supplementary Table S3. Assembly statistics for the *de-novo* assemblies of the three rivers.

	Aripo	Quare	Yarra	NCBI genome
**# scaffolds**	11,150	8,382	15,147	nd
**Longest scaffold (bp)**	16,773,556	22,850,742	10,339,082	nd
**Scaffold N50**	51	36	121	nd
**Scaffold L50 (bp)**	3,972,843	6,017,975	1,509,777	nd
**Scaffold N90**	334	194	992	nd
**Scaffold L90 (bp)**	135,571	362,480	37,420	nd
**% genome > 50kb**	92.13	93.87	89.38	nd
**Total length (bp)***	683,635,408	688,342,185	681,071,722	679,931,942
**% gaps (N)***	3.29	3.89	3.45	8.45
**BUSCO - Actinopterygii**				
**complete**	96.3	96.5	96.0	95.7
**single-copy**	94.3	94.4	93.5	93.8
**duplicated**	2.0	2.1	2.5	1.9
**fragmented**	1.4	1.2	1.6	1.7
**missing**	2.3	2.3	2.4	2.6

nd – not determined

* calculated on anchored linkage groups only

Supplementary Table S4. Distribution of male-linked SNPs by chromosome. SNPs for which all but 3 males (>66%) in each population are heterozygous and all females are homozygous were classified as male-linked.

	Aripo		Quare		Yarra	
**Chromosome**	**Down.**	**Up.**	**Down.**	**Up.**	**Down.**	**Up.**
1	87	118	208	56	53	205
2	118	58	243	62	52	327
3	81	49	194	32	10	111
4	68	58	170	45	10	52
5	54	61	197	46	92	265
6	84	154	89	99	24	89
7	122	139	162	80	163	247
8	35	53	101	604	42	323
9	94	39	208	76	21	199
10	132	119	244	85	121	172
11	46	75	120	28	116	82
12*	283	235	916	1504	561	4645
13	60	77	156	82	85	287
14	59	60	249	79	17	197
15	72	51	143	58	207	170
16	71	55	175	55	24	168
17	149	79	135	59	93	67
18	23	38	89	64	42	137
19	28	54	155	75	203	98
20	50	45	120	91	98	297
21	67	91	135	54	411	104
22	152	61	93	32	26	144
23	48	73	86	97	33	207

Down. – downstream population
Up. – upstream population
* sex chromosome

Supplementary Table S5. Average nucleotide diversity by population. Diversity was estimated as the proportion of segregating sites (Watterson's theta) in 50 kb windows and averaged across all windows.

		Genome	Sex chromosome
**Aripo**	**Downstream**	0.00360	0.00485
	**Upstream**	0.00237	0.00251
**Quare**	**Downstream**	0.00305	0.00387
	**Upstream**	0.00090	0.00163
**Yarra**	**Downstream**	0.00208	0.00287
	**Upstream**	0.00220	0.00234

Supplementary Table S6. Identified genes on putative Y-linked scaffolds. The total number of annotated genes was 196. Sixty proteins of unknown function are not represented in the table (this includes the 3 proteins of unknown function found in the Aripo watershed).

Gene name	Description	Count	River*	Genbank**	Chrom***
ADD3	Gamma-adducin	2	Ya	XM_017305457	1
adra1a	Alpha-1A adrenergic receptor	1	Qu	XM_008424673	12
AKNA	Microtubule organization protein AKNA	1	Qu	XM_008424637	12
ALAD	Delta-aminolevulinic acid dehydratase	1	Qu	XM_008424693	12
ARHGAP11A	Rho GTPase-activating protein 11A	1	Qu	XM_008397378	21
ARRDC1	Arrestin domain-containing protein 1	2	Qu	XM_008424643	12
Atp8b2	Phospholipid-transporting ATPase ID	2	Ya,Qu	XM_008424732	12
Aven	Cell death regulator Aven	1	Qu	XM_017302122	21
BARHL1	BarH-like 1 homeobox protein	1	Qu	XM_008424665	12
BNIP3L	BCL2/adenovirus E1B 19 kDa protein-interacting protein 3-like	1	Qu	XM_008424670	12
brcc3	Lys-63-specific deubiquitinase BRCC36	1	Qu	XM_008424562	12
C6	Complement component C6	2	Ya,Qu	XM_017307761	12
C7	Complement component C7	2	Ya,Qu	XM_008403687	unplaced
CACNA1A	Voltage-dependent P/Q-type calcium channel subunit alpha-1A	1	Qu	XM_017307837	12
Cacna1b	Voltage-dependent N-type calcium channel subunit alpha-1B	1	Qu	XM_017307828	12
CCDC152	Coiled-coil domain-containing protein 152	1	Ya	XM_008438664	20
ccnb1	G2/mitotic-specific cyclin-B1	1	Qu	XM_008424602	12
CCNG2	Cyclin-G2	2	Ya,Qu	XM_008424569	12
CCNI	Cyclin-I	2	Ya,Qu	XM_008424570	12
CEL	Bile salt-activated lipase	1	Qu	XM_017307734	12
CFAP44	Cilia- and flagella-associated protein 44	2	Ya,Qu	XM_008424558	12
CGNL1	Cingulin-like protein 1	1	Qu	XM_008402566	unplaced
Chek2	Serine/threonine-protein kinase Chk2	1	Ya	XM_017303285	unplaced
CHRM5	Muscarinic acetylcholine receptor M5	1	Qu	XM_008397381	21

COL1A2	Collagen alpha-2	1	Qu	XM_017307678	12
DAB2IP	Disabled homolog 2-interacting protein	1	Ya	XM_008402526	unplaced
daf-36	Cholesterol 7-desaturase	1	Qu	XM_008424701	12
dnajc25	DnaJ homolog subfamily C member 25	1	Ya	XM_017307726	12
DNM1	Dynamin-1	1	Ya	XM_017303277	12
DPYSL2	Dihydropyrimidinase-related protein 2	1	Qu	XM_017307911	12
efna5b	Ephrin-A5b	1	Qu	XM_008424718	12
Ehmt1	Histone-lysine N-methyltransferase EHMT1	1	Qu	XM_008424610	12
ENTPD2	Ectonucleoside triphosphate diphosphohydrolase 2	1	Qu	XM_017307733	12
entr1	Endosome-associated-trafficking regulator 1	1	Ya	XM_008402532	unplaced
fam160b2	Protein FAM160B2	2	Ya	XM_008402518	unplaced
fech	Ferrochelatase, mitochondrial	2	Qu	XM_008424594	12
FIBCD1	Fibrinogen C domain-containing protein 1	1	Ya	XM_017303287	unplaced
Fmn1	Formin-1	1	Qu	XM_008397379	21
G2E3	G2/M phase-specific E3 ubiquitin-protein ligase	1	Qu	XM_008424863	13
GFI1B	Zinc finger protein Gfi-1b	1	Qu	XM_008424660	12
GHR	Growth hormone receptor	2	Ya,Qu	XM_017307740	12
GLUL	Glutamine synthetase	1	Qu		
GRIN1	Glutamate receptor ionotropic, NMDA 1	1	Qu	XM_017307813	12
GSN	Gelsolin	2	Ya,Qu	XM_017307920	12
GSTT1	Glutathione S-transferase theta-1	1	Ya	XM_008402531	unplaced
GTF3C4	General transcription factor 3C polypeptide 4	1	Qu	XM_008424662	12
GTF3C5	General transcription factor 3C polypeptide 5	1	Qu	XM_008424702	12
htr1a-B	5-hydroxytryptamine receptor 1A-beta	2	Ya,Qu	XM_008424748	12
IGSF9B	Protein turtle homolog B	1	Qu	XM_017307737	12
KCNV2	Potassium voltage-gated channel subfamily V member 2	2	Ya,Qu	XM_008424587	12
Klk1b11	Kallikrein 1-related peptidase b11	1	Qu	XM_008424614	12
MAMDC4	Apical endosomal glycoprotein	1	Qu	XM_017307732	12

Man1b1	Endoplasmic reticulum mannosyl-oligosaccharide 1,2-alpha-mannosidase	1	Qu	XM_008424628	12
MFSD14A	Hippocampus abundant transcript 1 protein	2	Ya,Qu	XM_017307779	12
00000088	Similar to Actin, alpha cardiac	1	Qu	XM_008397375	21
00000094	Similar to Ryanodine receptor 3	1	Qu		
NARS1	Asparagine--tRNA ligase, cytoplasmic	2	Ya,Qu	XM_008424576	12
Niban2	Protein Niban 2	1	Qu	XM_008424642	12
Nim1k	Serine/threonine-protein kinase NIM1	2	Ya,Qu	XM_008424736	12
npr2	Atrial natriuretic peptide receptor 2	2	Qu	XM_017307730	12
OARD1	ADP-ribose glycohydrolase OARD1	2	Qu	XM_017303228	unplaced
ONECUT2	One cut domain family member 2	1	Ya	XM_008424565	12
Os07g0515000	tRNA wybutosine-synthesizing protein 2/3/4	1	Qu	XM_017307777	12
Parm1	Prostate androgen-regulated mucin-like protein 1 homolog	1	Qu	XM_008424751	12
PHPT1	14 kDa phosphohistidine phosphatase	1	Qu		
PIGO	GPI ethanolamine phosphate transferase 3	1	Qu	XM_008424720	12
PPP2R2A	Serine/threonine-protein phosphatase 2A 55 kDa regulatory subunit B alpha isoform	1	Qu	XM_017307735	12
pum3	Pumilio homolog 3	2	Ya,Qu	XM_017307725	12
RAB27B	Ras-related protein Rab-27B	3	Ya,Qu	XM_008424581	12
RAPGEF1	Rap guanine nucleotide exchange factor 1	1	Ya	XM_017303277	unplaced
rasgrf2	Ras-specific guanine nucleotide-releasing factor 2	1	Qu	XM_008423269	12
RYR3	Ryanodine receptor 3	2	Qu	XM_017302123	21
Scg5	Neuroendocrine protein 7B2	1	Qu		
Sh3bp2	SH3 domain-binding protein 2	2	Ya,Qu	XM_008403810	unplaced
SHROOM3	Protein Shroom3	4	Ya,Qu	XM_017307728	12
SOWAHB	Ankyrin repeat domain-containing protein SOWAHB	2	Ya,Qu	XM_008424573	12
SPAG8	Sperm-associated antigen 8	1	Qu	XM_017307982	12
SPIN1	Spindlin-1	1	Ya	XM_017306593	9

SPINW	Spindlin-W	1	Ya		
SPINZ	Spindlin-Z	1	Qu		
Ssna1	Sjoegren syndrome nuclear autoantigen 1 homolog	1	Qu	XM_008424608	12
ST8SIA3	Sia-alpha-2,3-Gal-beta-1,4-GlcNAc-R	1	Ya	XM_008424566	12
STOM	Erythrocyte band 7 integral membrane protein	1	Qu	XM_017307919	12
Stoml2	Stomatin-like protein 2, mitochondrial	1	Qu	XM_008424721	12
Stxbp1	Syntaxin-binding protein 1	1	Qu	XM_008424639	12
Taf1c	TATA box-binding protein-associated factor, RNA polymerase I, subunit C	1	Qu	XM_008424568	12
Tbx3	T-box transcription factor TBX3	1	Qu	XM_008403160	unplaced
TCF12	Transcription factor 12	1	Qu	XM_008402569	unplaced
tmc2a	Transmembrane channel-like protein 2-A	1	Qu		
TMEM230	Transmembrane protein 230	1	Qu	XM_008424674	12
TRIM25	E3 ubiquitin/ISG15 ligase TRIM25	1	Qu	XM_008424560	12
Trim29	Tripartite motif-containing protein 29	3	Ya,Qu	XM_008434801	17
TSC1	Hamartin	1	Qu	XM_008424661	12
Txnl1	Thioredoxin-like protein 1	3	Ya,Qu	XM_008424578	12
UNC13B	Protein unc-13 homolog B	2	Qu	XM_017307753	12
Unc5d	Netrin receptor UNC5D	1	Qu	XM_017307716	12
VLDLR	Very low-density lipoprotein receptor	2	Ya,Qu	XM_008424754	12
Wdr7	WD repeat-containing protein 7	2	Ya,Qu	XM_017307727	12
Whrn	Whirlin	2	Qu	XM_008424630	12
ZFAND5	AN1-type zinc finger protein 5	1	Qu	XM_008424601	12

* Qu: Quare; Ya: Yarra

** Genbank accession number of the best reciprocal BLAST hit to the NCBI reference genome annotation

*** Chromosome of the NCBI reference genome

Supplementary Figure S1. Inversion on the sex chromosome (Chromosome 12) between the reference genome (Künstner et al. 2016) and our female genome assemblies. a) Dot-plots of Chromosome 12 alignments between the reference genome and our three female assemblies. Forward alignments are drawn in blue and reverse alignments are drawn in red. The Aripo sequence shows a clear rearrangement of the first ~10 Mb in the NCBI reference involving an inversion and translocation to the middle of the chromosome. The Quare and Yarra assemblies did not have long enough scaffolds spanning the full length of the inversion, but inverted breakpoints (in red) are clearly visible at the same genomic positions of the NCBI genome as those found in Aripo. **b)** IGV snapshot around the inferred breakpoint positions in the NCBI reference. From read alignments, we identified clear breakpoints in all three female genomes for which the reads surrounding the breakpoints do not have the expected pair orientation (light blue – panel b)) and/or are soft-clipped at the breakpoint positions (panel c). **c)** Inferred structure of the genomic rearrangement between a target genome and the reference. The inverted segment is shown in red and approximate breakpoint positions are indicated. Arrows indicate the expected read-pair orientation of reads mapping near the breakpoints of both configurations.

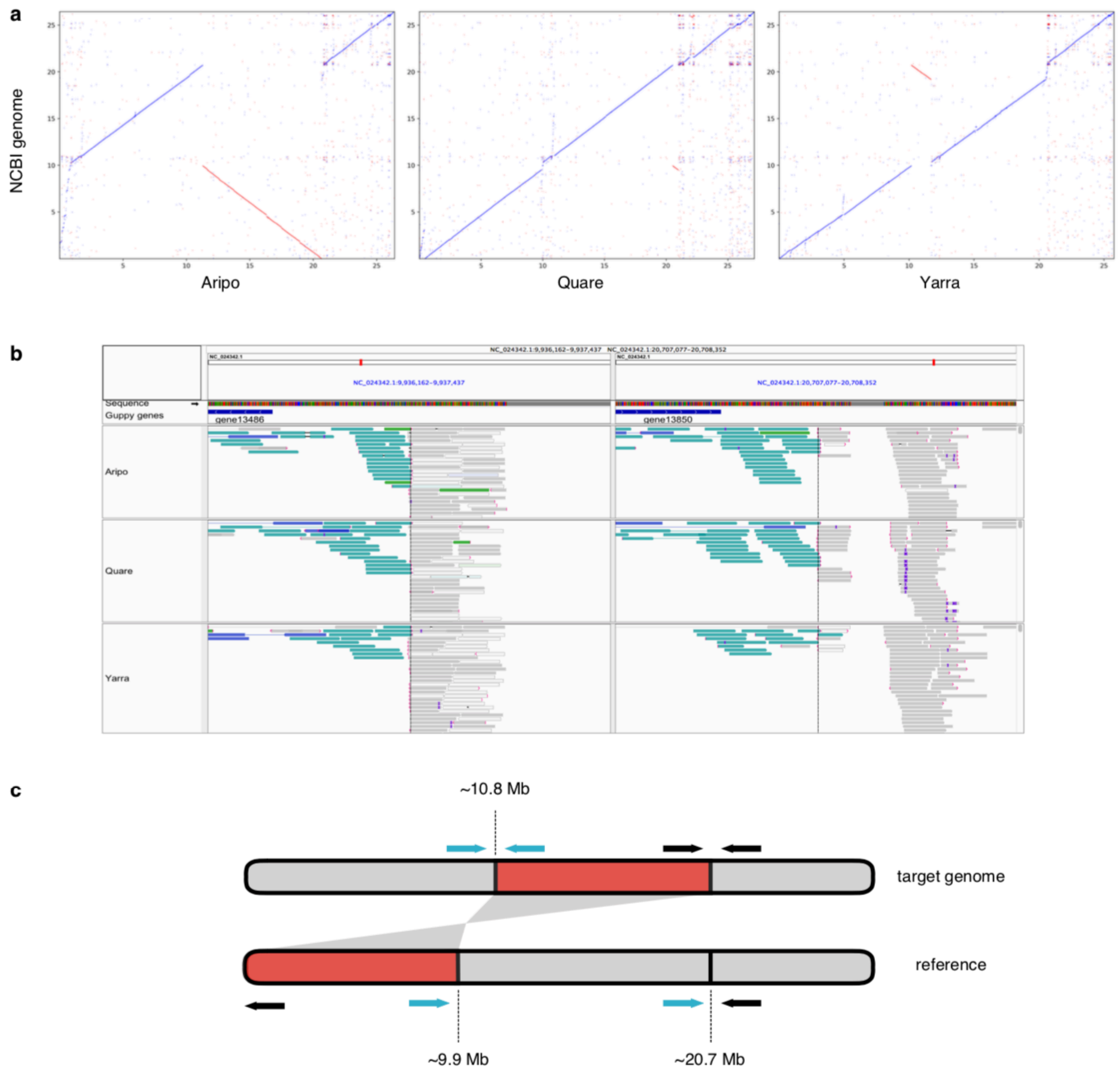

Supplementary Figure S2. Detection of the ancestral region of recombination suppression in the sex chromosome (Stratum I). a) Male to female coverage differences in Chromosome 12 for downstream, high-predation (black) and upstream, low-predation (orange) populations in the Aripo (**a**), Quare (**b**) and Yarra (**c**) watersheds. The $\log_2$ of male to female coverage was calculated in non-overlapping windows of 50 kb. The 95% confidence interval, inferred from bootstrapping autosomal regions is shaded in grey. The right-side density plots show the frequency (counts) of windows. ***, p-value < 0.001.

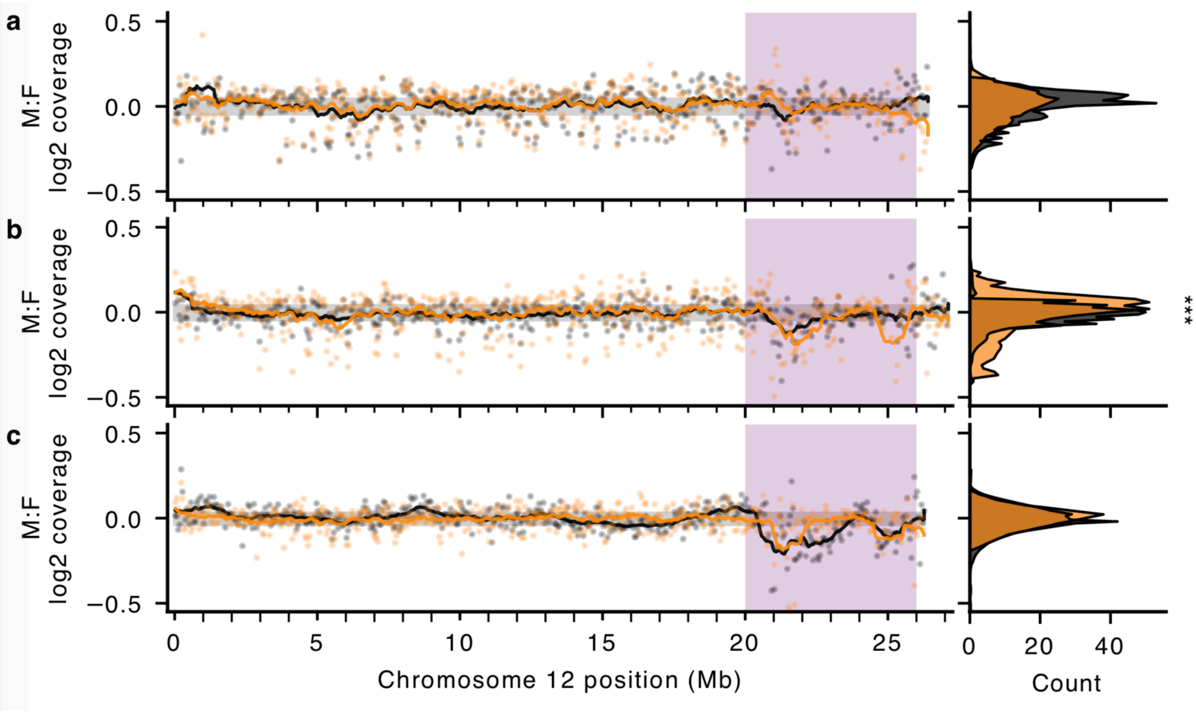

Supplementary Figure S3. Individual read coverage for Stratum I. Normalised median read coverage in non-overlapping windows of 50 kb. Coverage is plotted for each individual with males in blue and females in red.

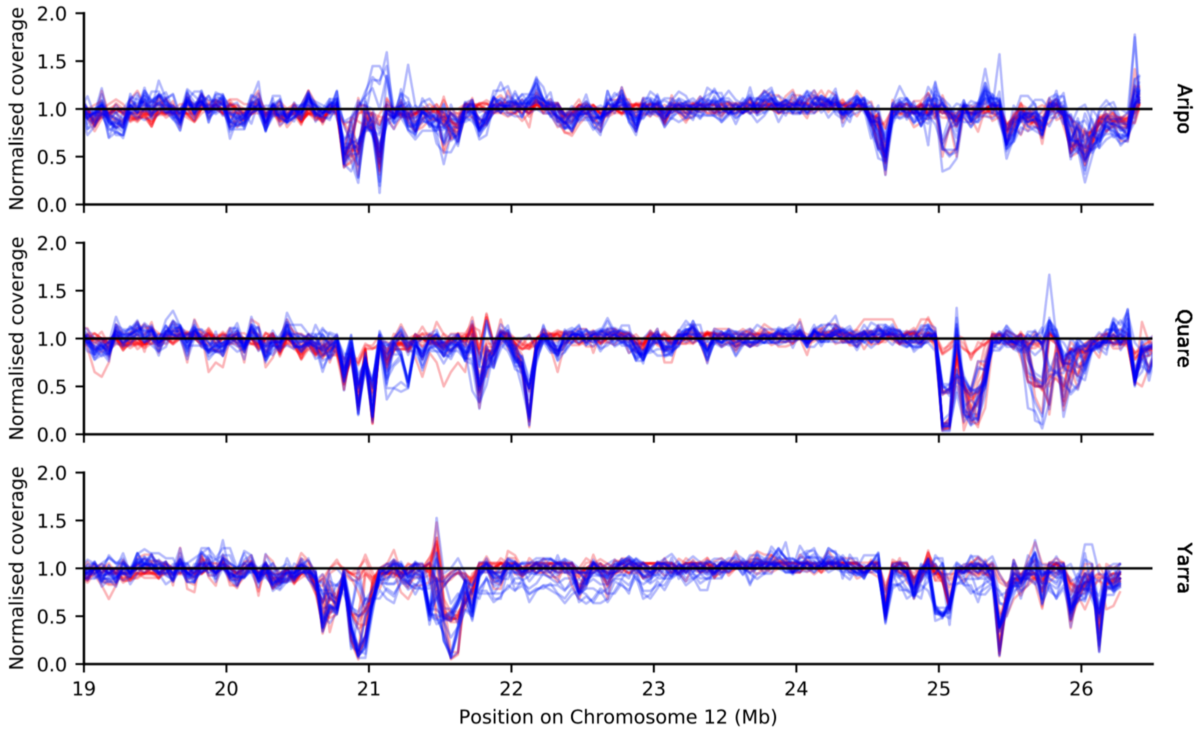

Figure S4. Evidence of incomplete lineage sorting in the pseudo-autosomal region (PAR) and Stratum I (SDR) of the sex chromosome. Principal components analysis (PCA) for all samples in the Aripo, Quare and Yarra watersheds. Panel **a** includes all SNPs on Chromosome 12 except for those found within Stratum I. Panel **b** includes all SNPs found within Stratum I. Males are shaded in blue and females are shaded in red.

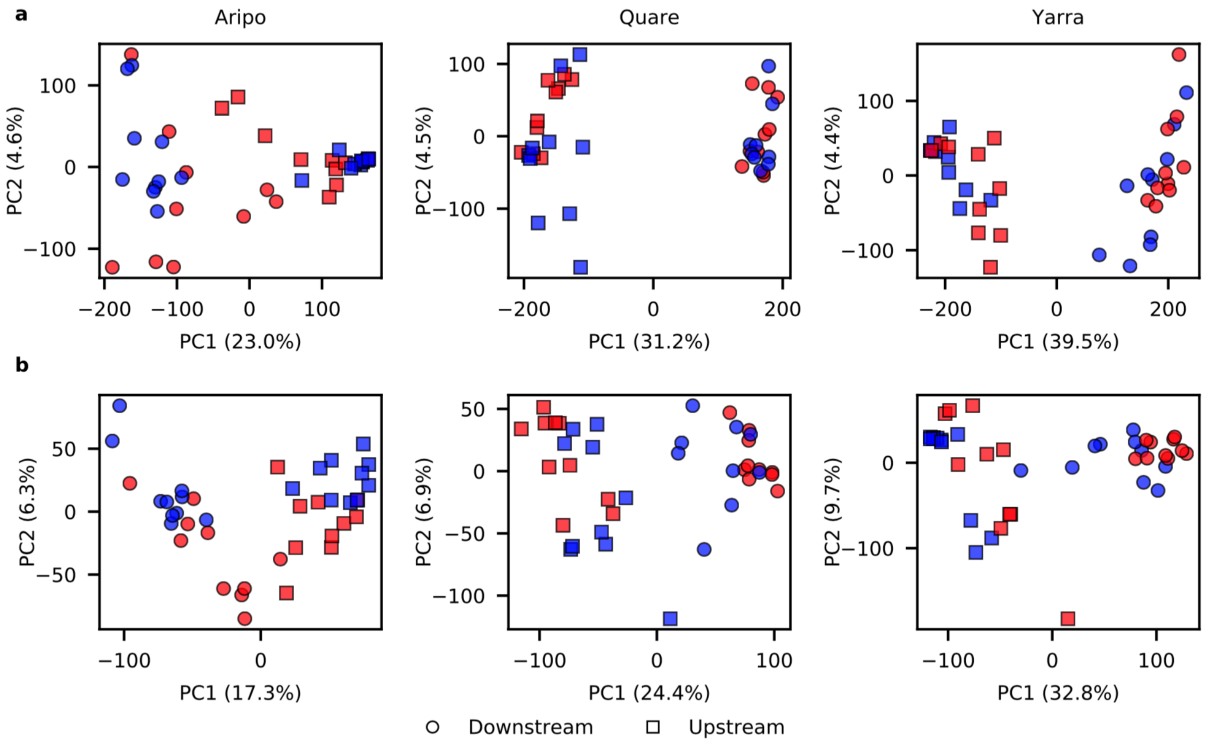

Figure S5. Long-range barcode overlap in males and females. 10x Genomics barcode overlap calculated in non-overlapping windows of 20 kb with all samples of each sex combined. The distribution of barcode overlaps is similar between males and females, suggesting that sex-chromosome divergence in the guppy is probably not driven by a large chromosomal rearrangement (e.g. inversion). **a-c)** Male plots in Aripo (a), Quare (b) and Yarra (c). **d-f)** Female plots in Aripo (d), Quare (e) and Yarra (f).

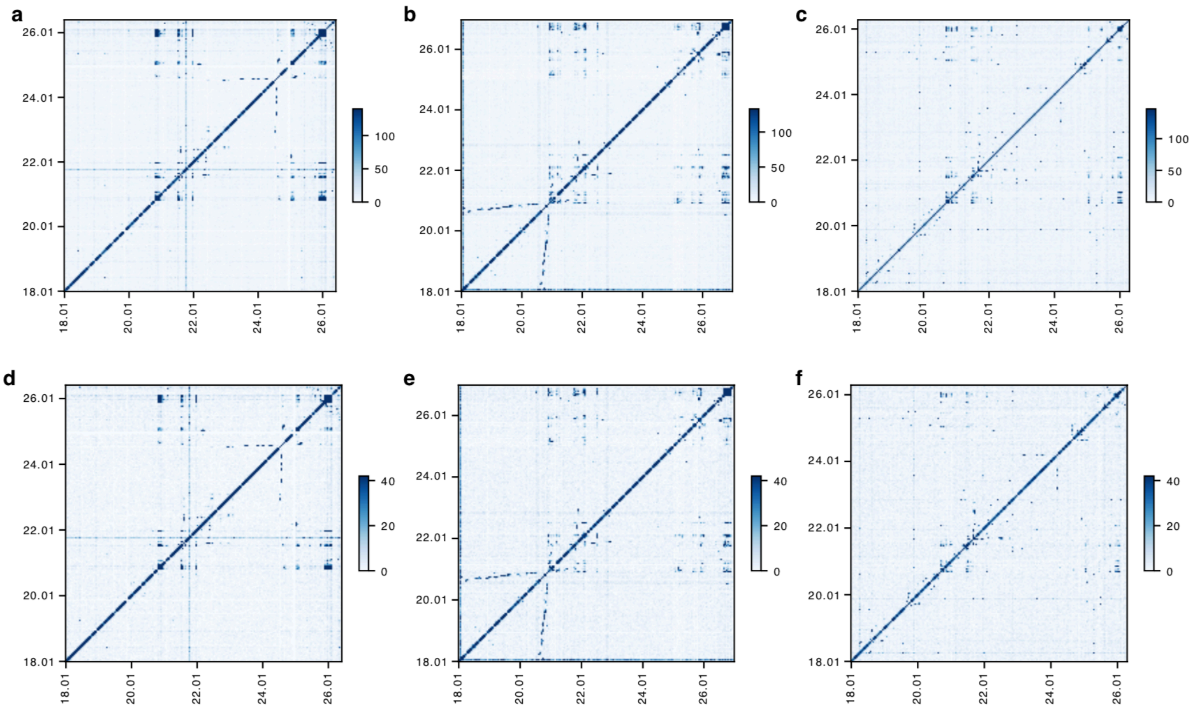

Supplementary Figure S6. Phase-switch error rate estimated for the samples sequenced with 10x Genomics. We calculated phase-switching errors after the computational phasing of all populations for the phase sets in each individual originally reconstructed with the 10x Genomics Long Ranger pipeline. The plots show the average phase switching (y axis) against the average phase length (x axis) per sample. Male samples are shaded in blue and females in red. The dotted line indicates a switching error rate of 5%.

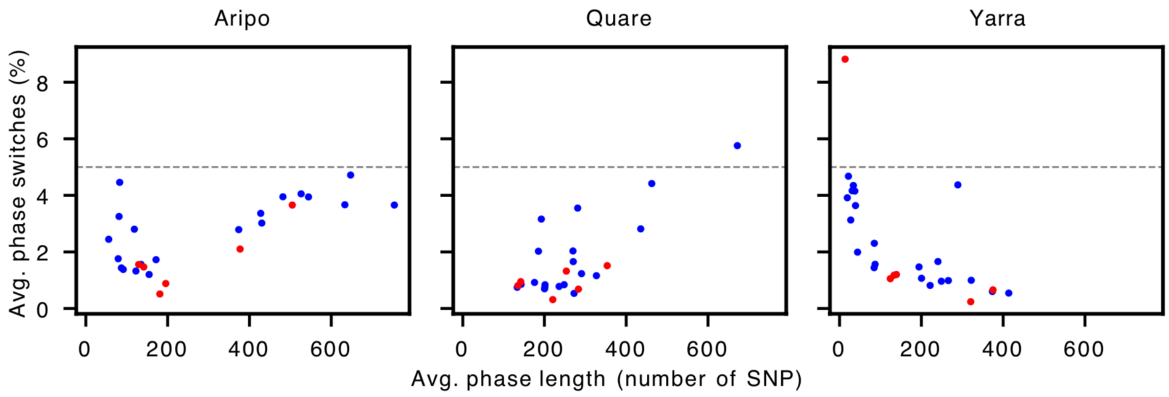

Supplementary Figure S7. Haplotype networks in the Quare River. Haplotypes identified in both upstream and downstream populations are shown. The circle area is proportional to haplotype frequency with the smallest circles representing single haplotypes, and branch lengths connecting haplotypes are proportional to the number of SNPs between haplotypes. Haplotypes from downstream, high-predation populations in grey and upstream, low-predation haplotypes in orange. The approximate location (in Mb) of each region on the sex chromosome is indicated above the network.

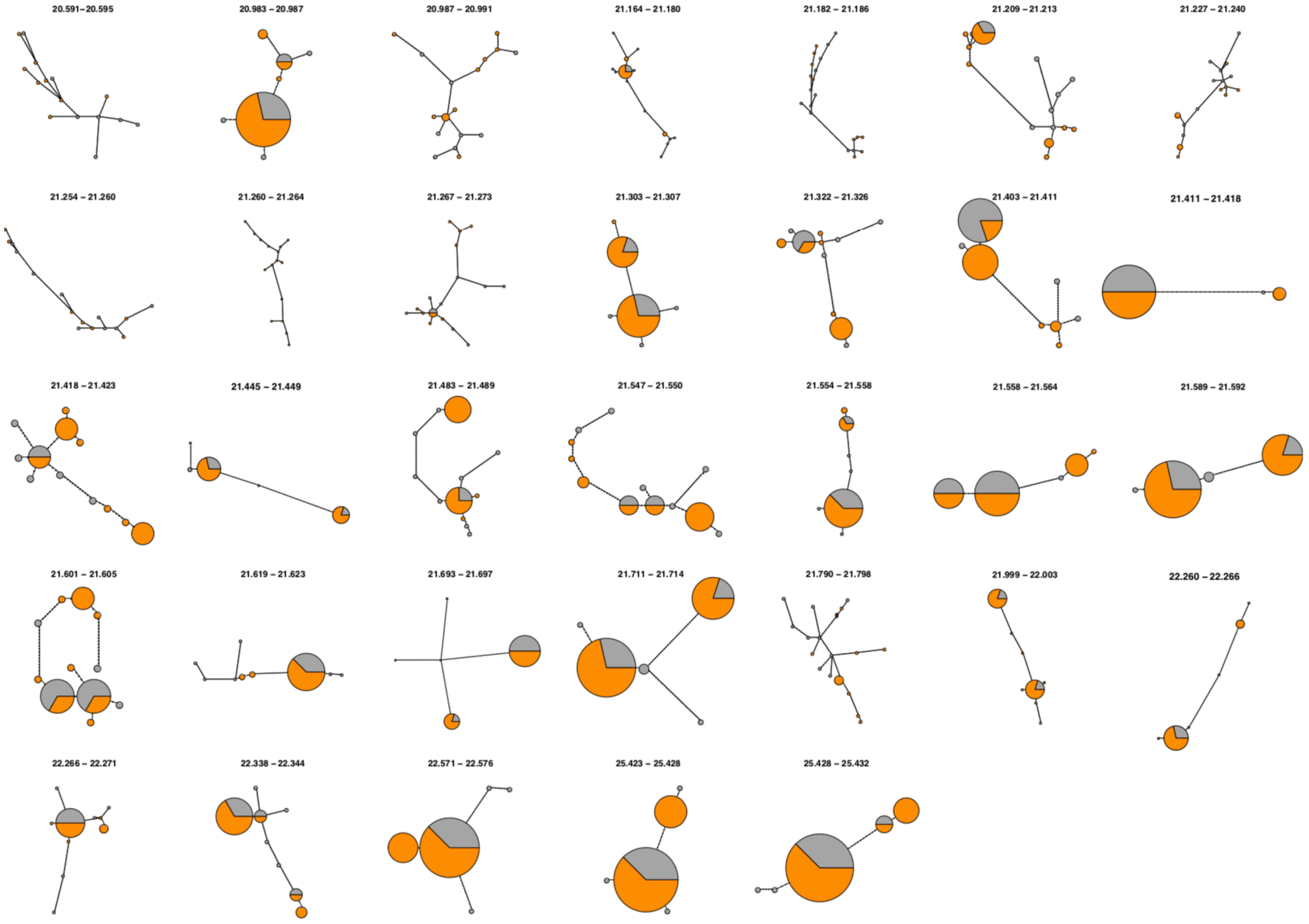

Supplementary Figure S8. Haplotype networks in the Yarra River. Haplotypes identified in both upstream and downstream populations are shown. The circle area is proportional to haplotype frequency with the smallest circles representing single haplotypes, and branch lengths connecting haplotypes are proportional to the number of SNPs between haplotypes. Haplotypes from downstream, high-predation populations in grey and upstream, low-predation haplotypes in orange. The approximate location (in Mb) of each region on the sex chromosome is indicated above the network.

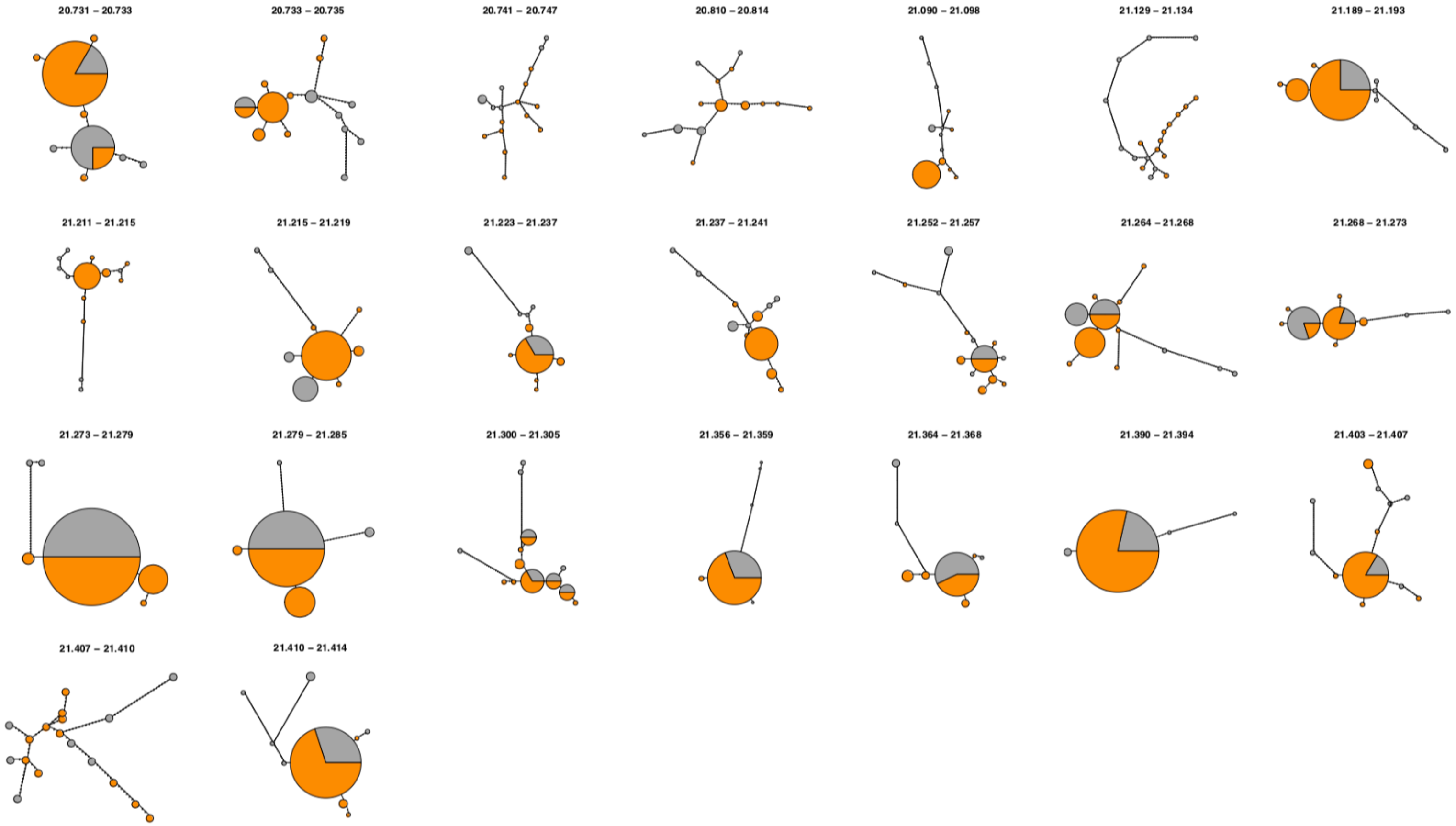

Supplementary Figure S9. False positive rate (FPR) in Y-mer discovery. a) Heatmaps of male- (M) and female- (F) specific k-mer counts in all populations. Numbers in the last column indicate the number of sex-specific k-mers fixed in each population. **b)** We used the FPR to estimate the frequency of a k-mer in the male population that can be confidently identified as a Y-mer. First, we recorded the total number of k-mers observed in only one sex at different frequencies in the population, between 1 and 10 individuals (a). Assuming that female-specific k-mers are false positives, the FPR of Y-mers was then estimated, at each frequency, as the number of female-specific k-mers relative to the number of male-specific k-mers minus female-specific k-mers. The dashed horizontal line indicates an FPR of 5%. Note that the slight increase in the FPR in the Quare-upstream population is lower than 5% (0.048).

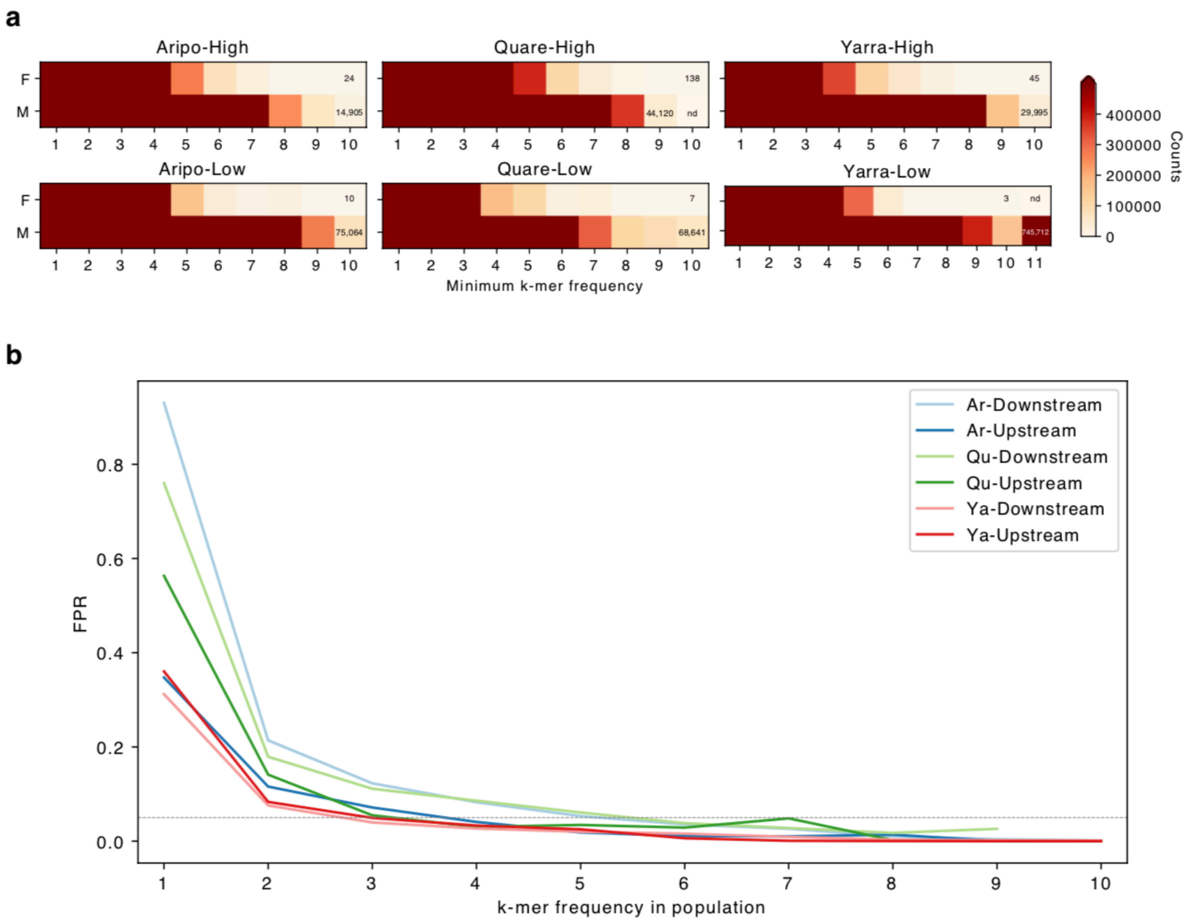

Supplementary Figure S10. Most Y-mers align non-uniquely to the genome. The barplots show the total percentage of Y-mers with unique and non-unique (repetitive) alignments to the respective reference genomes of each watershed. Given that Y-mers are male-specific by definition and the reference genomes come from females, we allowed for 1 difference, mismatch or gap, between Y-mers and the reference genome.

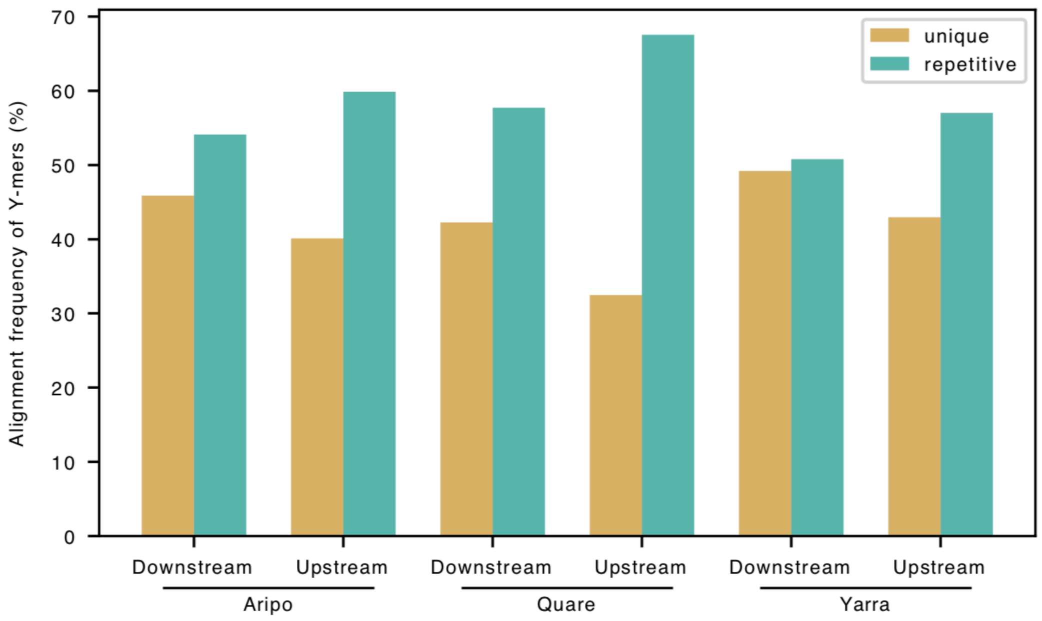

Supplementary Figure S11. Distribution of transposable elements (TEs) in the guppy sex chromosome. Boxplots showing the density (total sequence of TEs in every non-overlapping 50 kb window) of all classes of TEs (**a**), and for DNA transposons and Long Interspersed Nuclear Elements (LINEs) (**b**). Statistics were calculated for the Y-linked scaffolds (Y), the region of the X chromosome homologous to the SDR (X) and to the pseudo-autosomal region (PAR). ***p-value < 0.001, **p-value < 0.01, *p-value < 0.05.

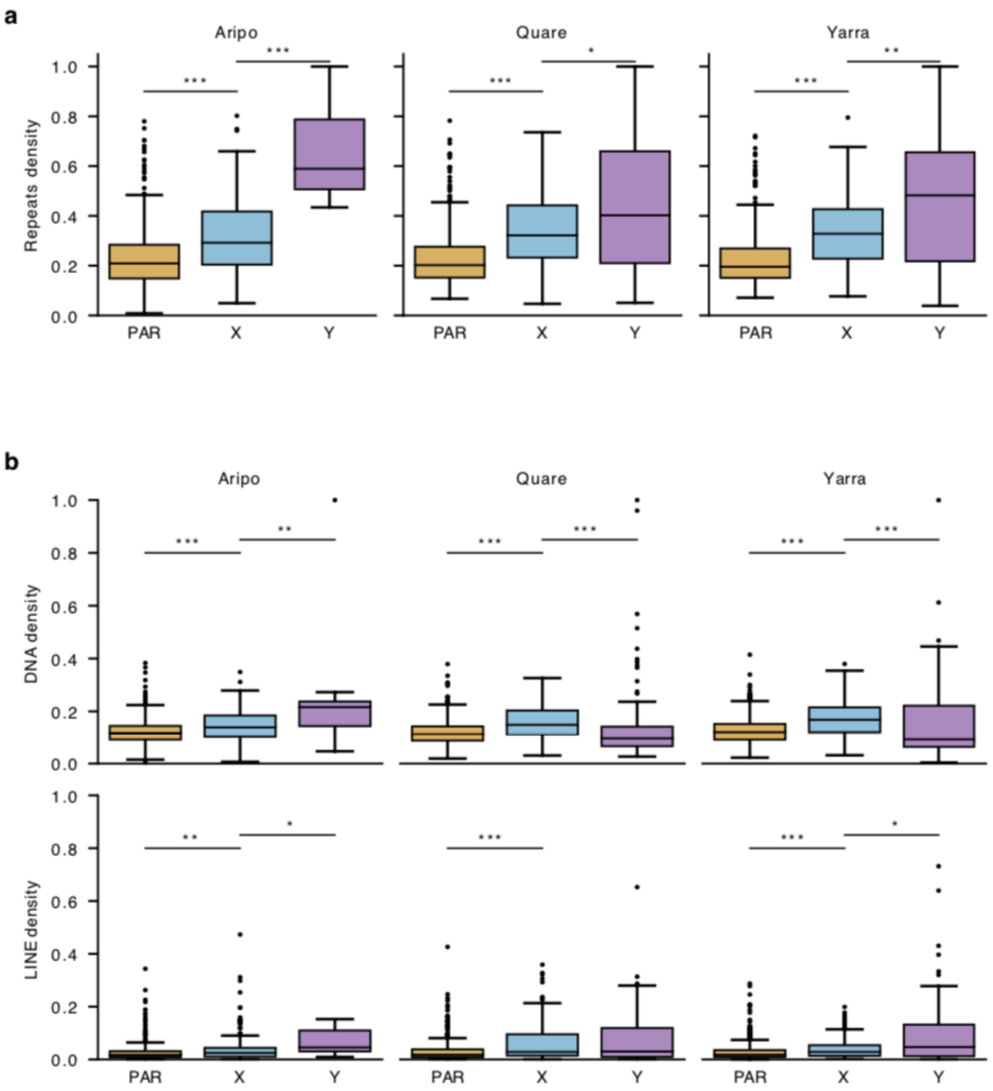

Supplement: msaa257_Supplementary_Data [file msaa257_supplementary_data.pdf]
